# Removing Gas from a Closed-End Small Hole by Irradiating Acoustic Waves with Two Frequencies

**DOI:** 10.3390/mi13010109

**Published:** 2022-01-10

**Authors:** Yuta Matsumoto, Yuki Mizushima, Toshiyuki Sanada

**Affiliations:** 1 Department of Engineering, Graduate School of Integrated Science and Technology, Shizuoka University, 3-5-1 Johoku Naka-ku Hamamatsu Shizuoka, Hamamatsu 432-8561, Japan; matsumoto.yuta.17@shizuoka.ac.jp; 2 Department of Mechanical Engineering, Faculty of Engineering, Shizuoka University, 3-5-1 Johoku Naka-ku Hamamatsu Shizuoka, Hamamatsu 432-8561, Japan; mizushima.yuhki@shizuoka.ac.jp

**Keywords:** multiphase flow, small hole, acoustic wave, natural frequency, gas–liquid interface oscillation, droplet, bubbles

## Abstract

Filling microstructures in the air with liquid or removing trapped gases from a surface in a liquid are required in processes such as cleaning, bonding, and painting. However, it is difficult to deform the gas–liquid interface to fill a small hole with liquid when surface tension has closed one end. Therefore, it is necessary to have an efficient method of removing gas from closed-end holes in liquids. Here, we demonstrate the gas-removing method using acoustic waves from small holes. We observed gas column oscillation by changing the hole size, wettability, and liquid surface tension to clarify the mechanism. First, we found that combining two different frequencies enabled complete gas removal in water within 2 s. From high-speed observation, about half of the removal was dominated by droplet or film formation caused by oscillating the gas column. The other half was dominated by approaching and coalescing the divided gas column. We conclude that the natural frequency of both the air column and the bubbles inside the tube are important.

## 1. Introduction

Cleaning, painting, and bonding using liquids are indispensable in product manufacturing. To completely achieve these processes, the inside of surface holes must be filled with liquid [1,2,3]. This is easy in a through-hole or larger diameter hole due to capillary action or Rayleigh–Taylor instability. However, it is difficult for liquid to enter small holes if one end is closed due to surface tension because it prevents gas–liquid interface deformation on a smaller, capillary-length scale [4,5,6]. Furthermore, gas dissolution with pressurization is no longer expected for large-aspect ratio holes because the gas volume becomes relatively large. Therefore, there is a need to remove gas from a closed-end hole efficiently. A general method is to apply a vacuum or steam to fill the closed-end hole with liquid. For example, Lin et al. demonstrated the vacuum filling of complex microchannels with liquid metal [7]. Unfortunately, these two methods require a pressure vessel and additional work such as pressurization and depressurization [8,9,10]. Thus, more efficient methods are required.

Sanada et al. [11] observed gas removal from a closed-end hole during a droplet train impact. They saw that the pressure fluctuation during the droplet impact caused the gas column volume to oscillate inside the hole and that the droplet formation from the oscillating gas–liquid interface promoted gas removal. The repeatedly generated droplet deposition split the gas in the hole. Furuya et al. [12] proposed using acoustic wave irradiation in a liquid to remove gas. They showed that releasing part of the gas at specific frequencies (*f* = 600 Hz) when irradiating with a single-frequency sine wave achieved complete gas removal when the frequency increased monotonically with time. They classified the gas removal process into three stages: in the first, gas removal and air column division occur; in the second, the divided air columns oscillate; and third, the divided air columns coalesce and are removed. The authors showed that the frequencies of the first and third stages are important for complete gas removal. However, the detailed gas removal mechanism is still unknown, so they conducted limited experiments. Further experiments are therefore needed on such factors as different hole diameters and the wettability of liquids other than water.

The purpose of this study is to elucidate the mechanism of gas removal. First, to find the optimum frequency for gas removal from closed-end holes we focused on the importance of two stages for complete gas removal from closed-end holes and attempted a faster removal by irradiating sinusoidal waves with two frequencies. Next, we observed gas removal by the irradiation of acoustic waves on closed holes of different diameters in water or ethanol and explored the most important factor of the first stage. Finally, we observed a more detailed mechanism, especially the final stage of the gas removal, and found the important factor for the second stage. We showed that the natural frequency of both the air column and the divided bubbles inside the hole is important.

## 2. Experimental Setup and Conditions

### 2.1. Experimental Setup

**Figure 1:**
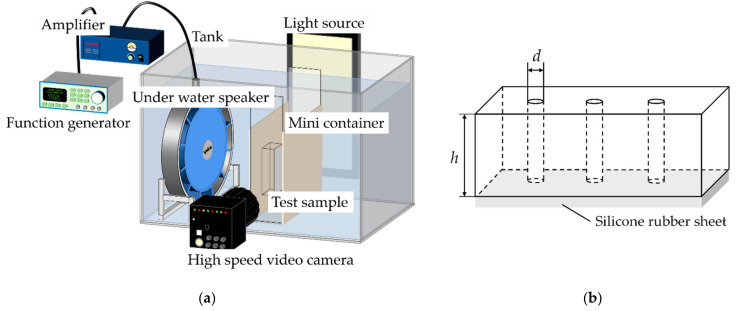
Experimental setup: (**a**) Schematic of experimental apparatus; (**b**) Schematic of the test sample.

Figure 1 shows a schematic of the experimental apparatus and test sample used in this study. In the device configuration (Figure 1a), we set an underwater speaker (UETAX, UA-30) in a water tank and installed a test sample in front of the speaker. A signal obtained by amplifying the sinusoidal wave output from the function generator (NF Corporation, WF-1974) with an amplifier (UETAX, UA-211) was used to drive the speaker. Figure 1b shows a test sample with three through-holes. We sealed the end of the holes with a silicon rubber sheet to make it closed ended. This experiment used the three test samples shown in Table 1 to observe the effect of hole size and surface wettability. The liquid was put into a small container, which helped us to observe the different liquids. The outer wall of the container was fixed 7 mm in front of the underwater speaker. The sample was fixed 5 mm from the inner wall of the container.

**Figure 2:**
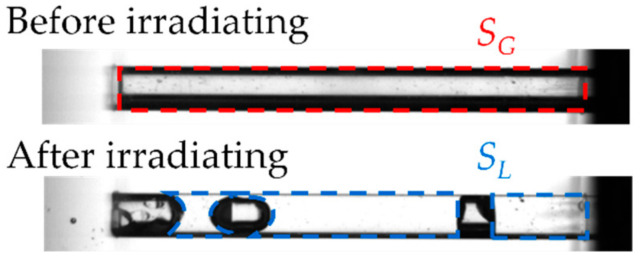
Definition of gas removal rate *r*. The gas column area *S*_G_ and liquid entering area *S*_L_ before and after irradiation.

We used a high-speed camera (Photron, FASTCAM Mini WX100) to observe the state of removing gas during acoustic wave irradiation. We used the gas removal rate *r* to evaluate the gas removal. We measured the gas column area *S*_G_ and liquid entering area *S*_L_ using MATLAB before and after irradiation by acoustic waves. Equation (1) shows the definition of *r*. Figure 2 shows a typical example of *S*_L_ and *S*_G_.

*r* = *S*_L_/*S*_G_ × 100 (1)

### 2.2. Experimental Procedure and Conditions

We conducted three tests (Table 2) to find the optimum gas-removal conditions and to clarify the mechanism. In test one, we irradiated two frequencies of sinusoidal waves to achieve complete gas removal from a closed-end hole initially filled with gas. A preliminary test (Furuya et al., 2019) clarified that irradiation at 600 Hz enhances removal; hence, we fixed the first stage frequency *f*_1_ = 600 Hz and irradiated for 1 s. Then, we varied the second stage frequency, *f*_2,_ from 1000 to 1500 Hz and irradiated for 1 s. In short, we tried to remove the gas completely in 2 s. We used sample one and filled a small container with water.

In test two, we investigated the gas removal of the first stage in detail. We irradiated a single-frequency sinusoidal wave for 1 s to the closed-end hole initially filled with gas. The frequency *f* was changed from 400 to 2000 Hz. We evaluated the gas removal rate and observed the removal. We used samples one, two, and three and filled a small container with water or ethanol.

In test three, we observed the removal process of the second stage. In test one, as described in Section 3.1 below, we found that the approaching and coalescing bubbles in the hole enhanced gas removal. Therefore, we prepared the break-up bubbles in the closed-end hole by inserting liquid using a micropipette and observed the irradiation of a single-frequency sinusoidal wave for 1 s at a frequency of 1100 Hz. For this test, we used sample one and filled a small container with water.

## 3. Results

### 3.1. Complete Gas Removal Condition (Test One)

**Figure 3:**
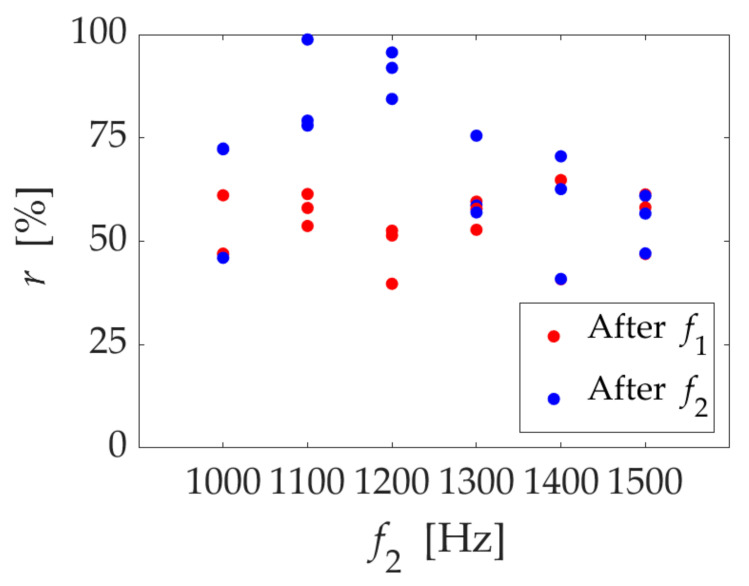
The gas removal rate *r* in two stages of irradiation. The red plot shows the first stage result after *f*_1_ = 600 Hz irradiation, and the blue plot shows the second stage result after *f*_2_ irradiation.

First, we fixed the first stage frequency (*f*_1_ = 600 Hz) and changed the second stage frequency *f*_2_ to find the perfect gas removal conditions. Figure 3 shows the results: the horizontal and vertical axes show *f*_2_ and the gas removal rate *r*, respectively. The red and blue plots show the result after *f*_1_ and *f*_2_ irradiation, respectively. Overall, *r* was about 50% after *f*_1_ irradiation. After the second irradiation, it increased at 1100 and 1200 Hz. In particular, we achieved complete gas removal with 1100 Hz irradiation. On the other hand, *r* hardly increased with *f*_2_ irradiation from 1300 to 1500 Hz.

**Figure 4:**
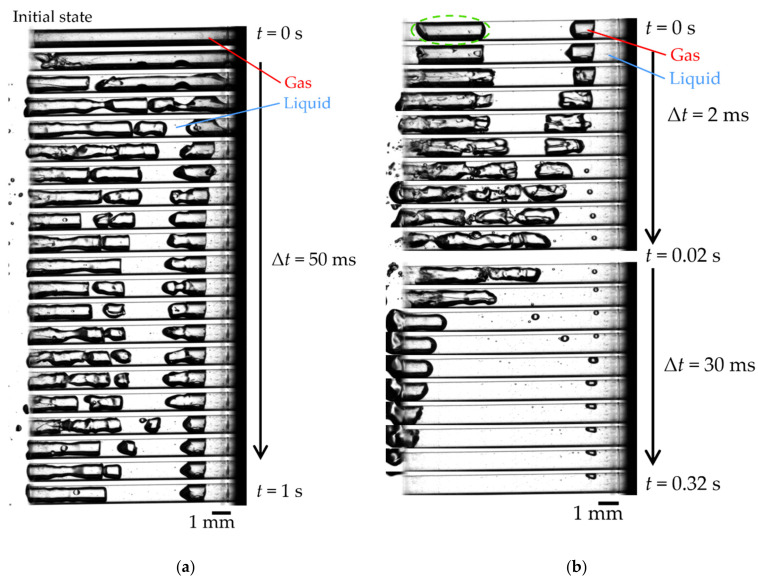
Visualization of gas removal process. The black stripes and the white areas are gas and liquid, respectively: (**a**) First stage irradiation (*f*_1_ = 600 Hz, Δ*t* = 50 ms). See  Supplementary Material Video S1 for a movie corresponding to Figure 4a; (**b**) Second stage irradiation (*f*_2_ = 1100 Hz). See Supplementary Material Video S2 for a movie corresponding to Figure 4b.

Figure 4 shows the visualization images of achieving complete gas removal (*f*_2_ = 1100 Hz in Figure 3). In Figure 4, the black stripes and the white areas are gas and liquid, respectively. Figure 4a shows the images when irradiated with first-stage *f*_1_ = 600 Hz. We confirmed that the air column was divided and part of the gas was removed simultaneously. Figure 4b shows images of the second stage after dividing the gas columns. As shown in Figure 4b, the right bubble gradually approached the other gas column in the early second stage (*t* = ~0.02 s) and coalesced. Then, the entire gas column was removed (*t* = 0.02–0.32 s). As shown in Figure 4, we confirmed that the two irradiation steps achieved complete gas removal in about 1.3 s.

### 3.2. First Stage Irradiation (Test Two)

**Figure 5:**
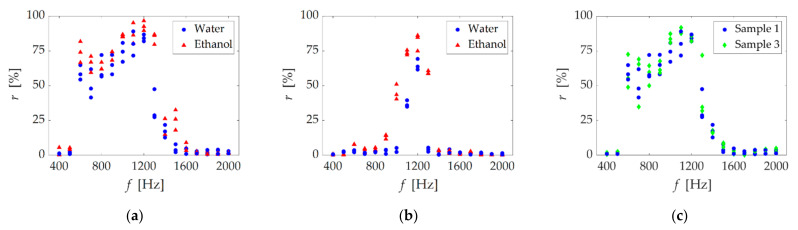
Gas removal rate *r* as a function of irradiate frequency *f*: (**a**) sample 1 (*d* = 1 mm), liquid surface tension effect; (**b**) sample 2 (*d* = 0.5 mm), hole size effect; (**c**) samples 1 (acrylic) and 3 (glass), material wettability effect.

Figure 5 shows the results of test two; single-frequency irradiation for different liquids (Figure 5a), hole size (Figure 5b) and hole material (Figure 5c). The horizontal and vertical axes show *f*, and *r*, respectively. In the single frequency irradiation, Figure 5a shows a high gas removal rate from 600 to 1300 Hz. Similarly, Figure 5b shows a high gas removal rate from 1000 to 1300 Hz. Furthermore, both Figure 5a,b show that *r* tended to be slightly higher in the case of ethanol than that of water. Note that, by carefully looking at Figure 5a, we find the two peaks at *f* = 600 and 1200 Hz. In contrast, Figure 5c shows almost the same trend for acrylic and glass holes, indicating that the material’s wettability had no effect.

### 3.3. Second Stage Irradiation (Test Three)

After the first stage of irradiation, several patterns of gas columns emerged. Depending on column division, the gas remained unremoved in the second stage. In test three, we examined the gas removal characteristics in the second stage under the following three initial situations, as shown in Table 3: (a) two bubbles remained, and liquid existed between the lower bubble and the bottom wall of the hole; (b) two bubbles remained and one bubble was attached to the bottom of the hole wall; that is, there was no liquid between the bubble and the wall); and (c) three bubbles remained.

**Figure 6:**
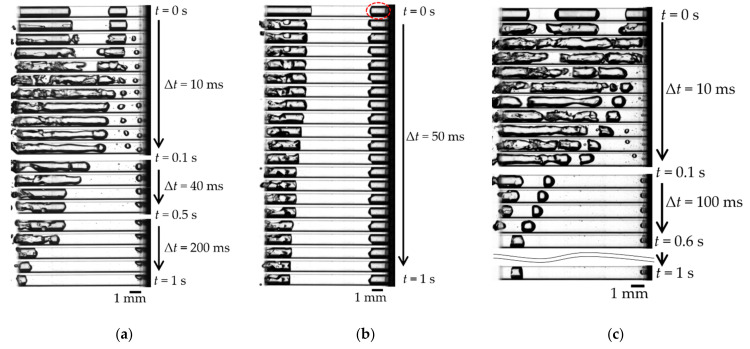
Gas removal process of second stage (*f* = 1100 Hz): (**a**) Condition (a). See Supplementary Material Video S3 for the movie corresponding to Figure 6a; (**b**) Condition (b). See Supplementary Material Video S4 for the movie corresponding to Figure 6b; (**c**) Condition (c). See Supplementary Material Video S5 for the movie corresponding to Figure 6c.

Figure 6 shows the second stage (*f* = 1100 Hz) of gas removal under the conditions presented in Table 3. In condition (a), we confirmed that two bubbles approached and coalesced similarly to that shown in Figure 4; then, the entire gas column was removed. In condition (b), the bubble near the hole entrance was removed; however, the bubble at the bottom of the hole (circled in red in Figure 6b) was not removed. Additional experiments with different frequencies also did not remove the bottom gas. In condition (c), even though one bubble was attached to the bottom wall, three bubbles coalesced and were removed (Figure 6c).

## 4. Discussion

### 4.1. Complete Gas Removal Condition (Test One)

#### 4.1.1. Gas Removal Using Two Stages of Irradiation

We achieved complete gas removal in a short time by irradiating the two frequencies, as shown in Figure 3. Initially, the air column oscillated greatly in the first stage, and the gas was gradually released. We expected that the air column would be oscillated by the resonance. As shown in Appendix A, the air column’s natural frequency with *d* = 1 mm was about 600 Hz. The gas removal rate by *f*_1_ irradiation was about 50%, but it was not completely removed. We suspect that the broken-up bubble is no longer a single air column, and the size is outside the resonance frequency.

In the second stage, the broken-up bubbles oscillated greatly and were removed by irradiating different frequencies. We expected that resonance would occur again. We estimated the natural frequency of the bubble surrounded by the green circle in Figure 4 using bubble oscillation in a tube [13], then it estimated 995 Hz, which was close to the irradiation frequency of 1100 Hz. From test one, we considered that the air column’s resonance and the broken-up bubbles’ resonance are important for removing gas from a small closed-end hole.

#### 4.1.2. Air Column Break-Up Process

**Figure 7:**
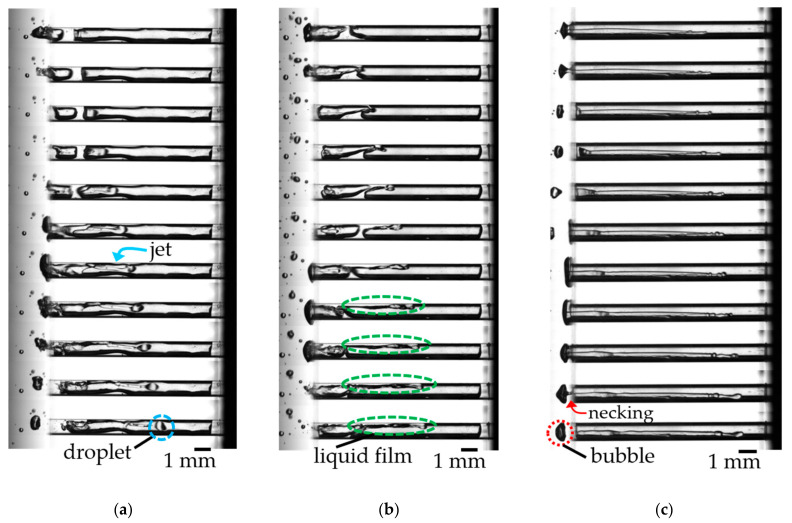
Visualized images of the gas removal mechanism (*f* = 600 Hz, Δ*t* = 0.2 ms): (**a**) Droplet generation. See Supplementary Material Video S6 for the movie corresponding to Figure 7a; (**b**) Liquid film formation. See Supplementary Material Video S7 for movie corresponding to Figure 7b; (**c**) Bubble generation. See Supplementary Material Video S8 for the movie corresponding to Figure 7c.

Next, we consider the air column break-up in the first stage. It seemed that this is the same mechanism as the liquid infiltration by the droplet train impingement shown by Sanada et al. [11]. Figure 7 shows detailed images of the gas removal process observed during acoustic irradiation. We show two key processes. The first is a droplet generation process shown in Figure 7a. As the gas shrank, the gas–liquid interface deformed, and the liquid entered the holes as a jet. The inertial force pinched off the infiltrating liquid and infiltrated into the hole as a droplet. The second is a liquid film formation shown in Figure 7b. The oscillation greatly deforms the gas–liquid interface, forming a liquid film between the wall and the gas. Additionally, then, the air column was broken-up by depositing the liquid in the holes from the above two factors.

We also confirmed the gas removal by the bubble break-up. The gas expanded to the outside of the hole during the air column oscillation, and the gas was pinched off from the necking point shown in Figure 7c. The bubbles that expanded outside the hole could not be returned into a small hole at the shrinking phase. Shirota et al. [14] discuss the role of the added mass of the surrounding liquid during bubble detachment from an orifice. We expect that the motion of the surrounding liquid during bubble oscillation, which plays an important role in estimating the natural frequency in Appendix A, also contributes to gas removal.

### 4.2. First Stage Irradiation (Test Two)

#### 4.2.1. Air Column Removal

Next, we discuss the first stage. As shown in Figure 5, sample one had a high gas removal rate from 600 to 1300 Hz and peaked at 600 and 1200 Hz. Sample two had a high gas removal rate from 1000 to 1300 Hz. As shown in Section 4.1.2, the three important factors for gas removal were (1) droplet generation, (2) liquid film formation, and (3) bubble generation. In particular, gas volume oscillation was very important for gas removal since the oscillation of the gas–liquid interface caused these factors. Mainly, the two following ways greatly enabled the oscillation of the air column: (1) the large sound pressure level from an irradiating acoustic wave, and (2) the matching the resonance of the air column. We considered the frequency at which the gas removal rate increased from both ways mentioned above.

**Figure 8:**
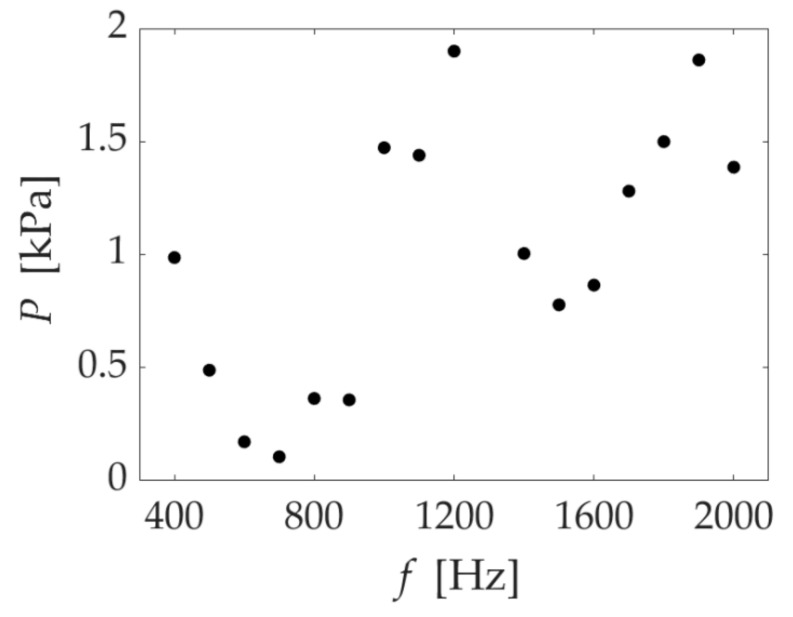
Frequency characteristics of underwater speakers.

In sample one, we confirmed two peaks in the gas removal rate at 600 and 1200 Hz. First, let us consider the first peak, 600 Hz. Figure 8 shows the frequency characteristics of the underwater speaker used in this study. Underwater speakers generally have large output changes depending on the frequency. We expected that the oscillation would increase in resonance at 600 Hz. At 600 Hz from Figure 8, the gas removal rate was high even though the sound pressure was low compared to the other frequency. In addition, 600 Hz was also consistent with the natural frequency of the estimated value (Appendix A). Next, we considered the second peak at 1200 Hz, which is twice the frequency of 600 Hz. We expected the gas removal rate to increase for the following two reasons: higher sound pressure and the resonance caused by the second harmonic. Figure 8 shows that the sound pressure at 1200 Hz was higher than the nearby frequencies. For 1900 Hz, the sound pressure was almost the same as that for 1200 Hz, but the gas removal rate was low. These results suggest that resonance by the second harmonic occurred at 1200 Hz. Note that the removal rate was greater for only 600, not 1200, Hz for speakers with different acoustic characteristics [12]. For these reasons, we observed two peaks in sample one.

In sample two, the gas removal rate peaked at 1200 Hz and we expected air column resonance to occur. The frequency of the high gas removal rate was approximately the same as the estimated value of the air column’s natural frequency (Appendix A).

#### 4.2.2. Effect of Physical Properties

The ethanol case slightly enhanced the gas removal rate in Figure 5a,b. We considered that bubble break-up and droplet generation were likely to occur in ethanol, a low surface tension liquid, which deformed the gas–liquid interface easily, and caused pinching of droplets to occur frequently. The contact angle was also small; therefore, ethanol easily spread on the wall surface to form a liquid film. From the above discussion, we determined that ethanol promoted gas removal.

In contrast, the surface wettability hardly affected the removal rate from Figure 5c. Liquid spread easily as a liquid film on the inner glass wall, which had better wettability compared to acrylic. This result indicated that droplet or bubble generation was dominant compared to the liquid film formation.

### 4.3. Second Stage Irradiation (Test Two)

#### 4.3.1. Oscillation and Movement of Break-Up Bubbles (Condition (a))

**Figure 9:**
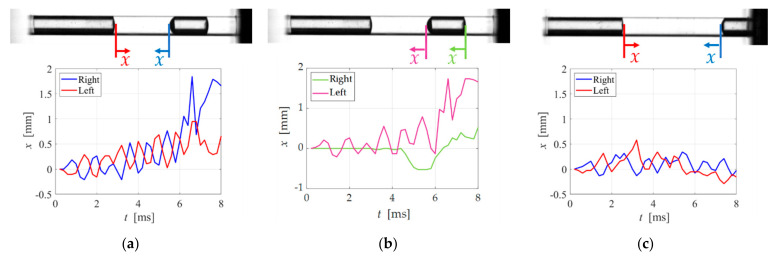
Displacement by interfacial oscillation (*f* = 1100 Hz): (**a**) Two adjacent bubbles (condition (a)). See Supplementary Material Video S9 for the movie corresponding to Figure 9a,b; (**b**) Left and right of bubble (condition (a)); (**c**) Two adjacent bubbles (condition (b)). See Supplementary Material Video S10 for the movie corresponding to Figure 9c.

Finally, we discussed the oscillation and movement of the break-up bubbles confirmed in test three. First, we focused on the approaching motion of bubbles near the hole bottom to another bubble near the hole entrance. Figure 9a shows the displacement *x* of the interface between the two bubbles from the initial state. We expected the two interfaces would oscillate in the same phase because the wavelength of the acoustic pressure was about 1.5 m, which is larger than the distance between the two bubbles. However, the interfaces of the bubbles oscillated in the opposite phase. In other words, when the interface on the left side expanded, the interface on the right shrank, and vice versa. This result suggested that the bubble near the hole bottom was oscillated by the bubble near the hole entrance. Therefore, *x* increased as the two bubbles approached, as shown in Figure 9a.

Supporting data for the hole bottom bubble oscillated by the bubble near the hole entrance were the differences in the oscillation of the left and right interface of the bottom bubble as shown in Figure 9b. The interface on the right-side of the bubble hardly oscillated until *t* = 4 ms, when even the left interface oscillated. We considered that the right side of the bubble was first pinned to the wall. Then, the right-side interface oscillations gradually wetted on the wall surface. Finally, the entire bubble moved to the left side. Note that no effect of the wettability described in the previous section was valid for only the first stage and may have affected the weak oscillating bubble at the bottom of the second stage.

#### 4.3.2. Moving and Unmoving of Bottom Bubbles: Condition (b) and (c)

Some bubbles remained unmoved, regardless of the entrance bubble oscillation, i.e., condition (b), as shown in Figure 6b. Figure 9c shows the interface displacement *x* of the two bubbles from the initial state. The oscillation amplitude at the right bubble interface of Figure 9c was smaller than that of Figure 9a. We expected that the oscillation of the left bubble would be attenuated by distance before being transmitted to the bottom bubble. In contrast, in the case of condition (c) of Figure 6c, i.e., the three-bubble case, the bottom bubble moved and was finally removed. The bottom bubble oscillated because of the oscillation of the center bubble, which was oscillated by the entrance bubble oscillation. We determined that the oscillation was propagated to neighboring bubbles so that the interface of the bottom bubble could be oscillated by the close distance between the bubbles. The large deformation of the interface caused the movement of the bubbles achieved their removal, as shown in Section 4.3.1. From the discussion mentioned above, we concluded that oscillating the bubbles near the hole entrance was important for removing the divided gas column (bubble). In other words, irradiating the natural frequency of the entrance bubble was efficient for achieving gas removal.

## 5. Conclusions

We demonstrated the removal of gas from a closed-end small hole by irradiating acoustic waves with two frequencies. Although liquid with lower surface tension tended to remove the gas more easily, the first stage of irradiation removed much of it at the natural frequency of the gas column, which was determined by the size and depth of the hole. After this stage, the gas column was divided into a few bubbles. In the second stage, approaching and coalescing bubbles dominated the gas removal. The asymmetric oscillation of the bubble due to the presence of the wall caused this approaching motion. We concluded that the resonance of the air column and the break-up bubbles were important for gas removal from the closed-end hole using acoustic wave irradiation.

## Figures and Tables

**Table 1:** Test sample shape and material.

	*d* [mm]	*h* [mm]	Material	Contact Angle [°]
Sample 1	1	10	Acrylic	75.6
Sample 2	0.5	5	Acrylic	75.6
Sample 3	1	10	Glass	43.0

**Table 2:** Experimental condition.

	Sample	Liquid	Irradiated Frequency [Hz]
Test 1	Sample 1	Water	*f*_1_ = 600, *f*_2_ = 1000~1500
Test 2	Sample 1, 2, 3	Water, Ethanol	*f* = 400~2000
Test 3	Sample 1	Water	*f* = 1100

**Table 3:** Initial test three conditions for gas column separation.

	Model	Visualized
Condition (a)	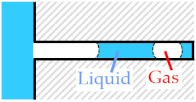	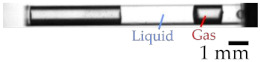
Condition (b)	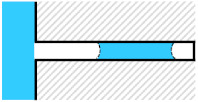	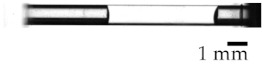
Condition (c)	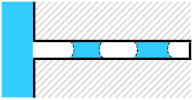	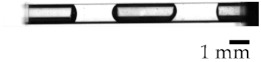

**Figure A1:**
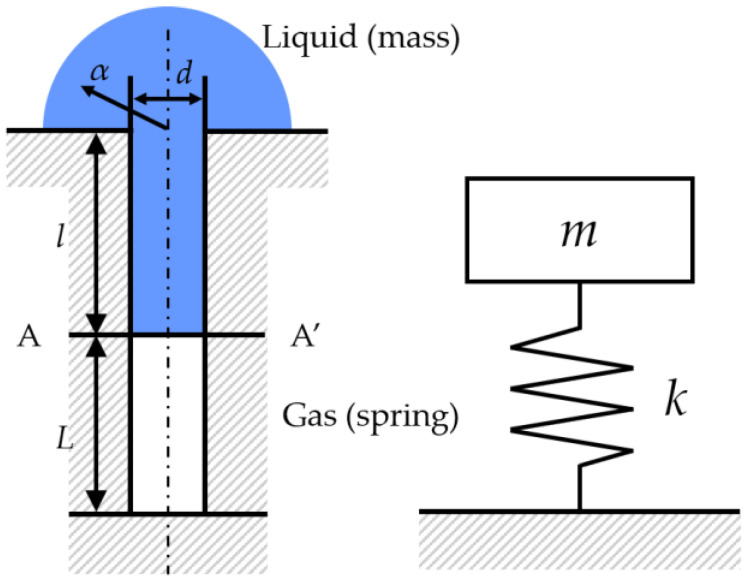
Gas column oscillation model.

**Figure A2:**
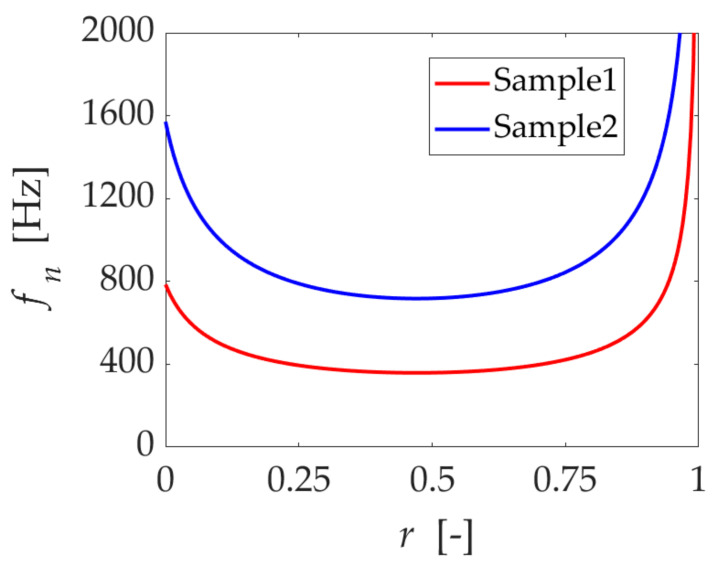
Relationship between the estimated natural frequency of a gas column *f*_n_ and gas removal rate *r*.

## Data Availability

All experimental data are available upon reasonable request from the corresponding authors via email.

## Supplementary Materials

The following supporting information can be downloaded at: https://www.mdpi.com/article/10.3390/mi13010109/s1, Video S1: *f*_1_ irradiation, playback speed 0.1 times., Video S2: *f*_2_ irradiation, playback speed 0.1 times, Video S3: Gas removal under condition (a), playback speed 0.1 times, Video S4: Gas removal under condition (b), playback speed 0.1 times, Video S5: Gas removal under condition (c), playback speed 0.1 times, Video S6: Gas removal mechanism-droplet infiltration, playback speed 3 × 10^−3^ times, Video S7: Gas removal mechanism-liquid film formation, playback speed 3 × 10^−3^ times, Video S8: Gas removal mechanism-bubble break-up, playback speed 3 × 10^−3^ times, Video S9: Interfacial oscillation of break-up bubbles under condition (a), playback speed 3 × 10^−3^ times, Video S10: Interfacial oscillation of break-up bubbles under condition (b), playback speed 3 × 10^−3^ times.

## Appendix A. Natural Frequency for a Gas Column

Furuya et al. [15] proposed a natural frequency for a gas column. Here, we introduce the model. The spring (spring constant *k*) and mass *m* system has a natural frequency *f_n_* expressed as follows:

(A1) $$f_{n}=\frac{1}{2}\pi \sqrt{\frac{k}{m}}.$$

The gas inside the hole and surrounding liquid are assumed to be spring and mass, respectively, as shown in Figure A1. We evaluated *k* and *m* as follows. We assumed that the gas and liquid were in contact at A–A′, and a polytropic process held during gas compression. The pressure changes before *P*_0_ and after compression *P* with movement *x* is as follows:

(A2) $$\frac{P}{P_{0}}=\frac{L^{\gamma }}{{\left(L-x\right)}^{\gamma }}\approx 1+\frac{x}{L}.$$

where *γ* is the polytropic constant. Therefore, *k* is expressed by Equation (A3):

(A3) $$k=\frac{\left(P-P_{0}\right)\pi }{x}{\left(\frac{d}{2}\right)}^{2}=\frac{\gamma \pi P_{0}}{L}{\left(\frac{d}{2}\right)}^{2}.$$

We assumed that the flow was a potential flow, and the flow outside and inside of the hole were a source-sink and uniform flow, respectively. The kinetic energy of the uniform flow inside the hole *E_hole_* is expressed by Equation (A4):

(A4) $$E_{hole}=\frac{1}{2}\rho V_{hole}u^{2}=\frac{\rho \pi u}{2}{\left(\frac{d}{2}\right)}^{2}\left(l+\frac{d}{3}\right).$$

where *u* is the uniform velocity inside the hole, *V_hole_* is the liquid volume inside the hole, and *l* is the liquid column length. The source-sink flow energy outside the hole *E_out_* is expressed with radial velocity *u_r_* by the integral of the range *R* (Figure A1),

(A5) $$E_{out}=\frac{1}{2}\rho V_{out}u_{r}{}^{2}=\frac{1}{2}\rho \int_{\frac{d}{2}}^{R}\frac{1}{2}4\pi \alpha^{2}u_{r}{}^{2}d\alpha \;.$$

Using the source-sink flow relation (*u_r_* = *Q*/2*πr*^2^), *R* is large enough, and *Q* should be the same as the flow from inside the hole; therefore,

(A6) $$E_{out}=\frac{\rho Q^{2}}{4\pi }\left(\frac{1}{d/2}-\frac{1}{R}\right)\approx \frac{Q}{2\pi d}=\frac{\rho \pi u^{2}}{4}{\left(\frac{d}{2}\right)}^{3}\;.$$

Hence, the entire kinetic energy *mu*^2^/2 is

(A7) $$E=\frac{\rho \pi u^{2}}{2}{\left(\frac{d}{2}\right)}^{2}\left(l+\frac{7}{12}d\right).$$

From Equation (A7), *m* is expressed as Equation (A8).

(A8) $$m=\rho \pi {\left(\frac{d}{2}\right)}^{2}\left(l+\frac{7}{12}d\right).$$

The obtained *k* (A3) and *m* (A8), and the natural frequency *f_n_* (A1) are estimated by Equation (A9),

(A9) $$f_{n}=\frac{1}{2\pi }\sqrt{\frac{12\gamma P_{0}}{\rho L\left(12l+7d\right)}}.$$

Figure A2 shows the relationship between the natural frequency *f_n_* of a gas column estimated from Equation (A9) and the gas removal rate, *r*, which is defined by Equation (A10),

(A10) $$r=\frac{l}{L+l}.$$

Here, we assumed the adiabatic process *γ* = 1.4.

## References

[1] Olim M. (1997). Liquid-Phase Processing of Hydrophilic Features on a Silicon Wafer. J. Electrochem. Soc..

[2] Spuller M.T., Hess W.D. (2003). Incomplete wetting of nanoscale thin-film structures. J. Electrochem. Soc..

[3] Vereecke G., Xu X., Tsai K.W., Yang H., Armini S., Delande T., Doumen G., Kentie F., Shi X., Simms I. (2014). Partial wetting of aqueous solutions on high aspect ratio nanopillars with hydrophilic surface finish. ECS J. Solid State Sci. Technol..

[4] De Gennes P.G., Brochard-Wyart F., Quéré D. (2004). Capillarity and Wetting Phenomena: Drops, Bubbles, Pearls, Waves.

[5] Ha J., Kim H.Y. (2020). Capillarity in soft porous solids. Annu. Rev. Fluid Mech..

[6] Daly B.J. (1969). Numerical study of the effect of surface tension on interface instability. Phys. Fluids.

[7] Lin Y., Gordon O., Khan M.R., Vasquez N., Genzer J., Dickey M.D. (2017). Vacuum filling of complex microchannels with liquid metal. Lab Chip.

[8] Horváth B., Kawakita J., Chikyow T. (2014). Through silicon via filling methods with metal/polymer composite for three-dimensional LSI. Jpn. J. Appl. Phys..

[9] Zhang Y., Yang B., Yang Z., Ye G. (2019). Ink-bottle effect and pore size distribution of cementitious materials identified by pressurization–depressurization cycling mercury intrusion porosimetry. Materials.

[10] Ko Y.K., Fuji H.T., Sato Y.S., Lee C.W., Yoo S. (2012). High-speed TSV filling with molten solder. Microelectron. Eng..

[11] Sanada T., Furuya Y., Muraki S., Watanabe M. (2018). Observation of liquid infiltration process into closed-end holes by droplet train impingement. J. Fluid Sci. Technol..

[12] Furuya Y., Mizushima Y., Watanabe M., Sanada T. (2020). Enhancement of Gas Discharge from a Closed End Hole by Using Acoustic Wave Irradiation. Jpn. J. Multiph. Flow.

[13] Oguz H.N., Prosperetti A. (1998). The natural frequency of oscillation of gas bubbles in tubes. J. Acoust. Soc. Am..

[14] Shirota M., Sanada T., Sato A., Watanabe M. (2008). Formation of a submillimeter bubble from an orifice using pulsed acoustic pressure waves in gas phase. Phys. Fluids.

[15] Furuya Y., Watanabe M., Sanada T. A model for a gas column oscillation inside a hole by irradiating an acoustic wave. Proceedings of the ASME-JSME-KSME Joint Fluids Engineering Conference.

